# In-Frame Deletion of Dystrophin Exons 8–50 Results in DMD Phenotype

**DOI:** 10.3390/ijms24119117

**Published:** 2023-05-23

**Authors:** Tatiana V. Egorova, Ivan I. Galkin, Oleg A. Velyaev, Svetlana G. Vassilieva, Irina M. Savchenko, Vyacheslav A. Loginov, Marina A. Dzhenkova, Diana S. Korshunova, Olga S. Kozlova, Dmitry N. Ivankov, Anna V. Polikarpova

**Affiliations:** 1 Laboratory of Modeling and Therapy of Hereditary Diseases, Institute of Gene Biology Russian Academy of Sciences, Moscow 119334, Russia; egorovatv@genebiology.ru (T.V.E.); i.simirina.marlin@gmail.com (I.M.S.);; 2 Marlin Biotech LLC, Sochi 354340, Russia; 3 Center for Precision Genome Editing and Genetic Technologies for Biomedicine, Institute of Gene Biology Russian Academy of Sciences, Moscow 119334, Russia; 4 Belozersky Institute of Physico-Chemical Biology, Lomonosov Moscow State University, Moscow 119991, Russia; 5 Core Facilities, Institute of Gene Biology Russian Academy of Sciences, Moscow 119334, Russia; 6 Center for Molecular and Cellular Biology, Skolkovo Institute of Science and Technology, Moscow 121205, Russia

**Keywords:** Becker muscular dystrophy, mouse model, DMD, BMD, dystrophin, genome editing, CRISPR–Cas9

## Abstract

Mutations that prevent the production of proteins in the *DMD* gene cause Duchenne muscular dystrophy. Most frequently, these are deletions leading to reading-frame shift. The “reading-frame rule” states that deletions that preserve ORF result in a milder Becker muscular dystrophy. By removing several exons, new genome editing tools enable reading-frame restoration in DMD with the production of BMD-like dystrophins. However, not every truncated dystrophin with a significant internal loss functions properly. To determine the effectiveness of potential genome editing, each variant should be carefully studied in vitro or in vivo. In this study, we focused on the deletion of exons 8–50 as a potential reading-frame restoration option. Using the CRISPR-Cas9 tool, we created the novel mouse model DMDdel8-50, which has an in-frame deletion in the *DMD* gene. We compared DMDdel8-50 mice to C57Bl6/CBA background control mice and previously generated DMDdel8-34 KO mice. We discovered that the shortened protein was expressed and correctly localized on the sarcolemma. The truncated protein, on the other hand, was unable to function like a full-length dystrophin and prevent disease progression. On the basis of protein expression, histological examination, and physical assessment of the mice, we concluded that the deletion of exons 8–50 is an exception to the reading-frame rule.

## 1. Introduction

Dystrophin deficiency induced by *DMD* gene mutations causes the progressive disorders Duchenne and Becker muscular dystrophies. On the basis of patient investigations, Monaco and coworkers presented a theory now known as the “reading-frame rule” [1] to explain differences in mutations underlying Duchenne muscular dystrophy (DMD, OMIM:310200) and Becker muscular dystrophy (BMD, OMIM:300376). Patients with DMD carry mutations that disrupt the translational open reading frame (ORF) and result in premature stop codons that entirely block the expression of the dystrophin protein. DMD is a severe type of the disease characterized by progressive muscle wasting, early loss of ambulatory function, and an estimated mortality rate of about 60% by age 25 [2]. Patients with BMD commonly have in-frame internal deletions that result in the expression of truncated but functional dystrophin proteins. Individuals with BMD experience a milder disease manifestation and progression and are able to walk till their senior years [3]. The “reading-frame rule” is supported by the majority of patient cases [4]. Nonetheless, there are exceptions to this principle. According to one study, one in five patients with large in-frame deletions will eventually acquire DMD [5]. Severe types of muscular dystrophy in patients with in-frame deletions in the *DMD* gene were related to mutation sites [5], dystrophin expression levels [6], or resultant dystrophin lengths [7]. The scientific community has not reached agreement on the fundamental qualities required for shortened dystrophin functionality, making it impossible to predict disease development in individuals with newly identified mutations.

The “reading-frame rule” is explained on a molecular level by a special dystrophin structure with a concentration of protein binding sites on its termini. There are four superdomains in full-length muscular dystrophin, Dp427m. Dystrophin binds to intracellular cytoskeleton proteins through the N-terminal actin-binding domain (N-ABD), which is made up of two calponin homology domains [8]. Mutations in N-ABD frequently cause DMD [9]. Twenty-four spectrin-like repeats (SR) make up the core domain, also known as the rod domain. This domain provides overall flexibility to dystrophin and mediates binding to some proteins. For example, SR11–15 present the actin-binding site, whereas SR16–17 create the nNos binding site [8].

Large deletions and other mutations in the rod domain are often well tolerated and frequently lead to BMD rather than DMD [10]. The cysteine-rich domain is required for the assembly of sarcolemmal dystrophin-associated glycoprotein complexes (DAGC) and is vital for dystrophin functioning [11]. Syntrophins and dystrobrevin binding are mediated by the C-terminal domain [12]. In the majority of the microdystrophin constructs that are now being investigated, this domain is typically absent, since it is relatively less important to maintaining basic dystrophin function [13]. Unstructured hinges separate the rod domain from the N-ABD and cysteine-rich domains. In the rod domain, there are two more hinges.

By eliminating exons from mature mRNA or genomic DNA, the exon-skipping approach aims to turn DMD into BMD. For individuals with the most frequent frameshift mutations in the central hotspot, antisense oligos-mediated exon-skipping is already being applied in the clinic after undergoing extensive testing in animal models [14]. The ability to skip up to 11 *DMD* exons was proven in vitro and in vivo in a mouse model [15]. With improvements in genome editing, reading-frame restoration is no longer limited to the exclusion of a single or a small number of nearby exons. Technically, removing one exon from gDNA is identical to removing ten exons [16,17]. Great expectations are placed on this approach, which is currently being investigated in animal models, as introduced changes are irreversible and permanent [18].

Gene editing therefore broadens the range of mutations that can be corrected with exon skipping to restore the reading frame. Concerns have been raised about the functionality of the resulting shortened proteins [19]. Contrary to in-frame deletions in the core mutation hotspot, which are common in BMD patients, large deletions in the *DMD* gene can have a significant impact on the domain architecture of the protein. For example, interdomain junctions and exon junctions may not always match up precisely [10]. The generated abnormal spectrin-like repeats may be nonfunctional or even detrimental to the protein’s structure [20], stability [21,22], and function [23]. Data from published studies [23] indicate that disturbance of SR phasing is the cause of early cardiomyopathy onset in BMD. Rational exon-skipping design has been successful in preventing the formation of aberrant or incomplete spectrin-like domains in a number of studies. The resulting dystrophin variants showed improved stability and provided a milder phenotype [17,24,25]. The importance of carefully assessing the functionality and stability of shortened dystrophin is highlighted by these productive experiments. However, it may not be enough to examine the functional capabilities of the resulting shortened versions in vitro and in silico. Therefore, BMD animal models that could illustrate the results of therapeutic genome editing are awaited.

Earlier, we identified a large, unique deletion of exons 8–34 of the DMD gene in a Russian DMD patient and created a patient-specific mouse model [26]. These animals suppressed the expression of Dp427m dystrophin due to frameshift mutation. The main characteristics of DMD have been demonstrated in generated mice. The histological, biochemical, and functional characteristics were comparable to those of the conventional *mdx* mouse model. The aim of this work was to assess the potential of a genome editing approach which excises additional exons to improve the phenotype caused by the deletion of exons 8–34. We offer a rational design of resultant deletion for this particular case, based on the relationship between the exon structure of the gene and the domain structure of the protein, and propose deletion of exons 8–50. However, the Leiden Open Variation Database contains information about a pathogenic in-frame deletion of exons 8–50 (ID #00286638) in a DMD patient. In order to define the genotype–phenotype correlation, we generated a mouse model with an in-frame deletion in the *DMD* gene and compared it to the previously described knock-out DMD model and healthy counterparts.

## 2. Results

### 2.1. Exons 8 to 50 of the DMD Gene Are Deleted Using the CRISPR-Cas9 Genome Editing Technique

**Figure 1:**
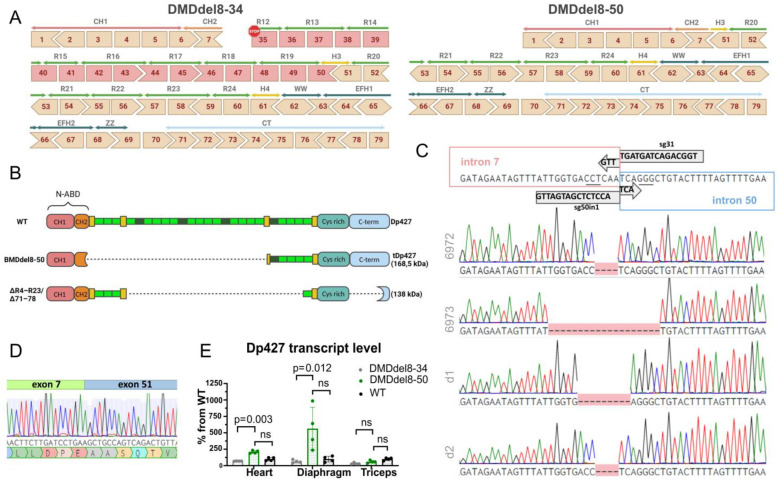
The generation of mice with in-frame deletion of exons 8–50 in the *DMD* gene by CRISPR-Cas9 genome editing. (**A**) The structure of the DMD gene with a deletion of exons 8–34 (left) and exons 8–50 (right). Exons and corresponding protein domains are shown. Calponin homology domains CH1 and CH2 make up the N-terminal actin-binding domain (N-ABD); R12-R24 are spectrin-like repeats within the central core domain; the cysteine-rich domain consists of subdomains WW (tryptophan-rich domain), EFH1, EFH2 (EF hand domains 1 and 2), and ZZ (zinc finger domain); the C-terminal domain (CT) is shown. The arrow shape of the adjacent exons indicates open reading frame (ORF) compatibility. Exons up to and including 50 must be removed to restore the ORF and are shown in red. (**B**) Domain structure of full-length dystrophin in wild-type mice (WT) and DMDdel8-50 mice bearing in-frame exons 8–50 deletion. For comparison, the structure of microdystrophin R4–R23/Δ71–78 [28] is shown. (**C**) Exact intron sequences of founder mice with deletion of exons 8–50. The scheme shows the theoretical sequence of intron 7 to intron 50 junction and the corresponding guide RNA-binding sites. PAM sites are underlined. (**D**) Transcript sequence at the junction of exons 7 and 51. Below is the corresponding protein sequence. (**E**) Comparison of Dp427 transcript levels in DMDdel8-34 and DMDdel8-50 muscle organs compared with wild-type animals. Quantitative RT-PCR was performed using primers to exons 3–5. A Kruskal–Wallis test with Dunn’s test for multiple comparisons was used. Data presented as mean ± SD. *p*-values are shown on the graph; ns—non-significant.

A significant part of the *DMD* gene’s protein-coding sequence is lost with deletion of exons 8 through 34. The remaining exons encode the N-ABD without the final 24 AA in the CH2 subdomain and the domains after SR12 in the rod (Figure 1A). We can restore the reading frame by deleting one or more exons using double-cut genome editing technology. The N-ABD plays a crucial role in the binding of dystrophin to the cellular cytoskeleton; hence, we chose to save all seven of the exons at the 5′ end. According to previous research, missense and in-frame mutations in this region cause DMD by provoking protein misfolding and instability, which interferes with the proper interaction with actin [27]. We chose a deletion of exons 8–50 as a preferred variant of reading frame restoration based on exon compatibility. Comparatively to a full-length protein, the resulting dystrophin molecule retains 39.5% of the amino acids. N-ABD connects to the core domain in full-length dystrophin via hinge 1. The resulting truncated dystrophin contains a 30 AA fragment of hinge 3 and intact spectrin-like repeats beginning with SR20 (Figure 1B). Binding sites for dystrophin partners, such as actin or DAGC components, are conserved, as are those in microdystrophins (Figure 1B) that are currently being tested for the treatment of DMD [13].

We used a double-cut CRISPR-Cas9 genome editing tool to remove about 945 kb from the *DMD* gene to generate a BMD model with deletion of exons 8 to 50. We utilized the previously described sg31 guide [26] to introduce the upstream double-strand break. We chose three guides from the CHOPCHOP online guides creation tool [29] for the downstream break; they are referred to as sg50in1-3 below (Table 1). Two of them (Figure S1) were chosen for zygote microinjections with sg31 and Cas9 mRNA because they performed better in vitro. After embryo transfer, we had 17 neonates, and 4 of them had the required deletion (Table 2). D1 and D2 died shortly after birth. All mice’s intron 7 to 50 junction site sequences corresponded well with the predicted scheme (Figure 1C). During the reparation, an extra 4–12 base pairs from intron 7 upstream of the double-strand break and 0–7 base pairs from intron 50 downstream of the cut site were eliminated. The second X-chromosome of female founder mouse 6972 underwent editing, which led to short deletions in both introns 7 and 50, according to guide target-site analysis (Table 3, Figure S2). In both of the X chromosomes of mouse 6973, however, the editing led to the fusion of introns 7 and 50.

In addition, we were unable to identify any editing activities in the top three predicted off-target locations for either guide (Figure S2). Exons 7–51 were correctly spliced after target exons 8–50 were deleted, restoring the reading frame (Figure 1D). Founders 6972 and 6973 were used for line derivation. We compared the resulting mouse model, hereafter referred to as DMDdel8-50, with both wild-type and DMDdel8-34 knockout (KO) mice. An in-frame transcript was found in the heart, diaphragm, and skeletal muscles of DMDdel8-50 mice (Figure S3). Reading-frame restoration should preserve the Dp427 transcript from nonsense-mediated decay. We used a qRT-PCR assay with primers to transcript the sequence preceding mutation, in order to compare Dp427 expression level. Indeed, in all investigated tissues, the Dp427 transcript level was equivalent to that of wild-type animals and higher than that of KO mice in the heart and diaphragm (Figure 1E).

### 2.2. In BMDdel8-50 Muscles, Dystrophin Expression Is Below Normal Levels

**Figure 2:**
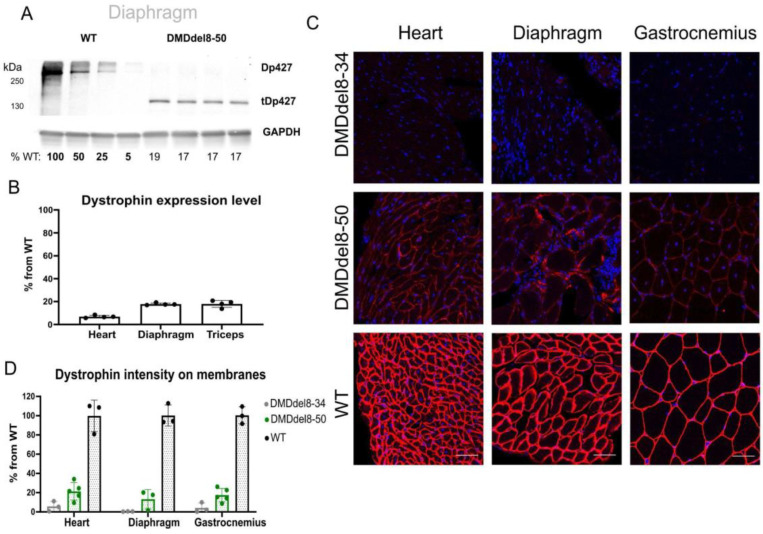
Truncated dystrophin expression in DMDdel8-50 mice does not reach wild-type levels. (**A**) Representative image of dystrophin expression analysis in triceps using a Western blot assay. KO: DMDdel8-34. Loading control: GAPDH. The total protein load per lane is shown below. (**B**) Dystrophin expression level normalized on wild-type muscle expression level. See also Figure S4B. (**C**) Immunofluorescence analysis of muscle transverse sections with antibodies against the C-terminus of dystrophin (Ab15277, red). Scale bar = 50 μm. (**D**) The intensity of immunofluorescence on the sarcolemma is normalized to the intensity of wild-type muscle staining. Data presented as mean ± SD. N = 3–4 animals per group. See also Figure S5.

The translation of the truncated Dp427m (tDp427m) transcript with the deletion of exons 8–50 results in shorter dystrophin, which has an estimated molecular weight of 168.5 kDa (Figure 1B). Using antibodies against the C-termini of dystrophin, we were able to detect matching protein bands (Figure 2A and Figure S4A). The expression of dystrophin was lower than it was in WT animals. We calculated that the average expression levels in the heart, diaphragm, and triceps were 6.8%, 17.8%, and 18%, respectively (Figure S4B and Figure 2B). In addition, we detected protein bands with a size between 70 and 100 kDa in the heart, diaphragm, and skeletal muscles of DMDdel8-50 animals. These protein products, which are absent in the muscles of wild-type and KO mice, most likely correspond to the dystrophin Dp71 variation (Figure S4A). The correct localization of dystrophin on the sarcolemma was discovered by immunofluorescence analysis of cross-sections of the heart, diaphragm, and gastrocnemius muscles (Figure 2C). The membrane staining intensity ranged from 10 to 21% of normal levels (Figure 2D).

### 2.3. Components of DAGC Are Diminished in BMDdel8-50 Muscles

**Figure 3:**
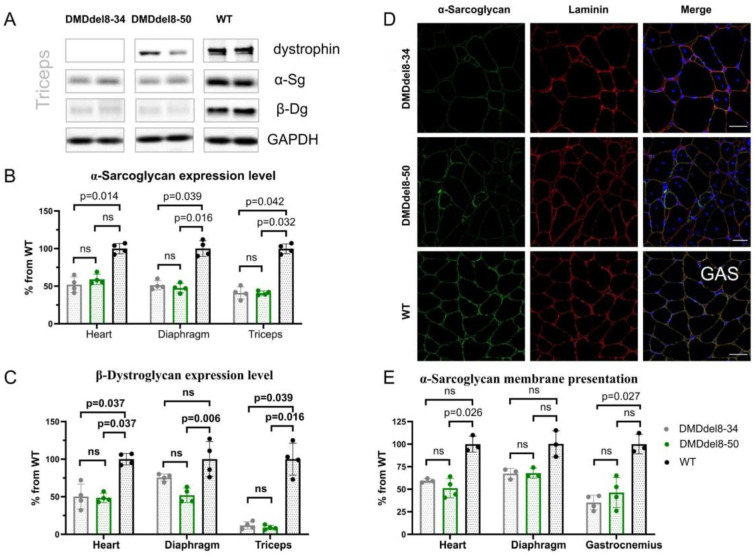
DAGC is not restored by truncated dystrophin expression. (**A**) Western blotting of DAGC components’ expression in the triceps. Loading control: GAPDH. (**B**) Quantitative analysis of Western blots for α-sarcoglycan expression in the heart, diaphragm, and triceps. See also S6A, left panel. (**C**) Quantitative analysis of Western blots for β-dystroglycan expression in the heart, diaphragm, and triceps. See also S6A, right panel. (**D**) Immunofluorescence analysis of gastrocnemius muscle sections with antibodies to α-sarcoglycan. Scale bar = 50 μm. (**E**) The intensity of α-sarcoglycan immunofluorescence staining on the sarcolemma. See also Figures S5 and S6B. A Kruskal–Wallis test with Dunn’s test for multiple comparisons was applied. Data presented as mean ± SD. N = 3–4 animals per group. *p*-values are shown on the graphs; ns—non-significant.

Examination of muscle biopsy samples taken from BMD patients frequently reveals a reduction in the expression of truncated dystrophin [30]. Usually, this is sufficient to halt the disease’s progression. Dystrophin can change the disease phenotype from DMD to BMD, even in the presence of minimal levels [31]. We initially looked at the recovery of DAGC components to determine the functioning of the shortened dystrophin protein. In comparison with KO mice, the Western blotting analysis of muscle lysates did not show an elevation in the amounts of α-sarcoglycan and β-dystroglycan in either of the muscular tissues (Figure 3A–C and Figure S3A). In the sarcolemma, we also did not notice any reconstitution of the DAGC component α-sarcoglycan (Figure 3D,E and Figure S3B). We observed some heterogeneity on cryosections of the gastrocnemius muscle from DMDdel8-50 mice, with a fraction of myofibers exhibiting a higher level of α-sarcoglycan expression (Figure 3D). However, this increase had no meaningful impact on the average level of membrane staining (Figure 3E). In the diaphragm and heart tissues, this pattern was less pronounced (Figure S6B). Thus, despite the shortened dystrophin’s proper location in the sarcolemma, DAGC was not restored.

### 2.4. Skeletal Muscles from BMDdel8-50 Mice Exhibit DMD-Like Characteristics

**Figure 4:**
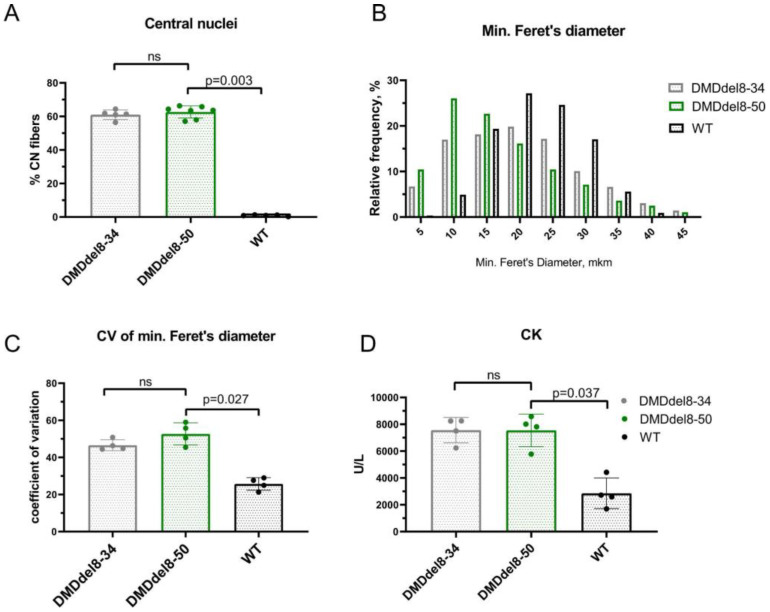
DMDdel8-50 mice’s skeletal muscles displayed signs of myofiber damage and regeneration. (**A**) Percentage of myofibers with central nuclei in gastrocnemius muscle. (**B**) Histogram of minimal Feret’s diameter of myofibers in gastrocnemius muscle. (**C**) Coefficient of variation of minimal Feret’s diameter of myofibers. (**D**) Serum creatine kinase level. A Kruskal–Wallis test with Dunn’s test for multiple comparisons was applied. Data presented as mean ± SD. N = 4–8 TA muscles (**A**–**C**), N = 4 mice (**D**). *p*-values are shown on the graphs; ns—non-significant. See also Figure 3D (merge) and Figure S7.

Skeletal muscles that lack dystrophin are more vulnerable to contraction-induced injury. Membrane damage causes muscle fiber necrosis and inflammation. Connective and adipose tissues replace necrotic muscle fibers. Myofibers mature from satellite cells and partially fill the necrotic areas during the regeneration process. They have nuclei that are positioned in the center and a smaller diameter. The percentage of myofibers with central nucleation is frequently used to track the progression of muscular dystrophy. Skeletal muscle histological changes in patients with BMD are extremely variable. More severely affected muscles may resemble DMD tissues, whereas pathology in less severely affected muscles may be limited to fiber size variation and mild fibrosis [32]. In the gastrocnemius muscle of 10-week-old DMDdel8-50 mice, we found an average of 63% of centrally nucleated myofibers, which is comparable to age-matched DMDdel8-34 mice muscles, but much higher than the content of normal muscles, which was just a small percentage (Figure 4A and Figure 3D, merge; Figure S7). Affected muscles typically contain large myofibers in addition to small ones, which raises the coefficient of variation of the myofibers’ diameter. In comparison to wild-type animals, we observed a leftward shift in the minimal Feret’s diameter and an increase in CV in DMDdel8-50 gastrocnemius muscle sections (Figure 4B and Figure S7). Little fibers (0–5 and 5–10 km intervals) were more common in DMDdel8-50 gastrocnemius sections than in wild-type animals or even DMDdel8-34 KO mice. Muscle enzymes are released into the blood when the integrity of muscle fibers is compromised, and, therefore, the level of creatine kinase in blood serum serves as an indicator for the progression of muscular dystrophy. We found elevated CK levels in DMDdel8-50 animals which were identical to those in KO mice (Figure 4E). Hence, in DMDdel8-50 mice, shortened dystrophin is unable to protect the skeletal muscle fibers from damage caused by contraction and the accompanying histological alterations.

### 2.5. Muscle Performance in DMDdel8-50 Mice Was Not Enhanced by Truncated Dystrophin

**Figure 5:**
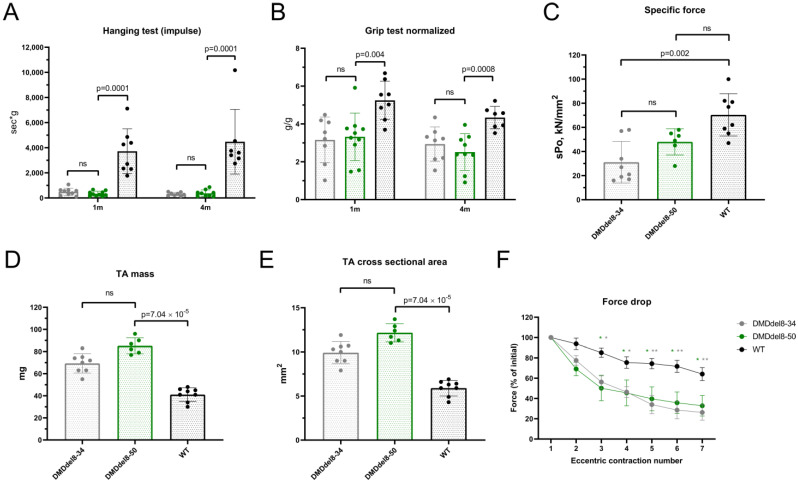
In juvenile and adult mice with exon 8–50 deletion, skeletal muscle function is compromised. (**A**) Maximal suspension time in hanging wire test of mice at 1 month and 4 months of age. (**B**) Grip force normalized to body weight. (**C**) Force generated by isolated TA muscle normalized on cross-sectional area. (**D**). TA muscle mass. (**E**) TA muscle cross-sectional area. (**F**) Reduction in TA muscle force generated in seven consequent eccentric contractions. An ordinary one-way ANOVA with Dunnett’s test for multiple comparisons was used for (**A**,**B**,**E**,**F**). A Kruskal–Wallis test with Dunn’s test for multiple comparisons was applied for (**C**–**E**). Data presented as mean ± SD (**A**–**E**) and mean ± SEM (**F**). N = 7–9 mice (**A**,**B**), N = 6–8 TA muscles (**C**–**F**). *p*-values are indicated on the graphs or marked * *p* < 0.05, ** *p* < 0.01; ns—non-significant.

We compared DMDdel8-50 mice with age-matched wild-type animals and KO DMDdel8-34 mice in a range of physiological tests to assess the functionality of truncated dystrophin. Performance in both juvenile and adult mice was evaluated using hanging wire tests and two-limb grip tests. The maximal hanging time was shorter than for WT mice at both time points and mirrored KO animals (Figure 5A). The dynamics of weight gain can vary depending on how a disease develops. A considerable degree of body weight fluctuation in the studied groups led us to normalize the parameter on mass, despite the fact that we did not discover a statistically significant difference in body weight between healthy and mutant animals (Figure S8). A two-limb grip test revealed that mice with an in-frame mutation had weaker muscles than their healthy counterparts. At both time points, grip strength was comparable to that of KO mice (Figure 5B). The final test, carried out on mice aged 6 months, involved eccentric contraction of the isolated tibialis anterior muscle. The specific force of the DMDdel8-50 muscle contraction was not substantially different from KO and wild-type groups, although the difference between KO and wild-type mice was noticeable (Figure 5C). DMDdel8-50 mice muscles were overweight compared to both wild-type and KO animals (Figure 5D), concealing the difference in the force produced during contraction (Figure S9). An increase in muscle cross-sectional area results from the replacement of myofibers with connective and adipose tissue. Intensive replacement in the patient’s muscles leads to calf pseudohypertrophy, one of the DMD features frequently observed. In-frame deleted mice had the tibialis anterior muscle cross-sectional area equivalent to that of KO animals. It was significantly larger than that of WT mice (Figure 5E). The reduction in force generated in repeated eccentric contractions was similar to that of KO muscles; both differed considerably from wild-type mice’s muscles, producing just 26 and 32% of the initial force at the seventh repetition, respectively, compared to 64% for healthy animals (Figure 5F).

### 2.6. DMDdel8-50 Dystrophin 3D Structure In Silico Prediction

We examined the 3D structure of the N-terminal segments of both the wild-type and the DMDdel8-50 variant dystrophins to better understand the DMD phenotype of the generated mouse model. The Protein Data Bank (PDB) lacks structural fragments of mouse dystrophin; however, some fragments of highly similar human dystrophin are available: two CH domains with a linker region between them in the dimeric open conformation (PDB: 1DXX), the first spectrin domain (PDB: 3UUN), and the WW domain (PDB: 1EG3). In order to comprehend the impact of exon skipping on the structure, we modeled the N-terminal region of DMDdel8-50 using AlphaFold.

The model included the first CH domain, the second CH domain’s N-terminal fragment, the hinge H3’s C-terminal segment, and spectrin repeat 20. We also modeled the mouse dystrophin N-terminal region, which included two CH domains, hinge domain H1, and the first spectrin repeat, to compare to the wild-type structure. AlphaFold predicted that the CH domains were in close conformation for both cases. To encourage the formation of the dimeric CH forms of the dystrophin observed in the PDB structure 1DXX, we added an additional chain of the N-terminal part of the sequence up to the hinge domain. As a result, the predicted structure adopted the dimeric conformation.

**Figure 6:**
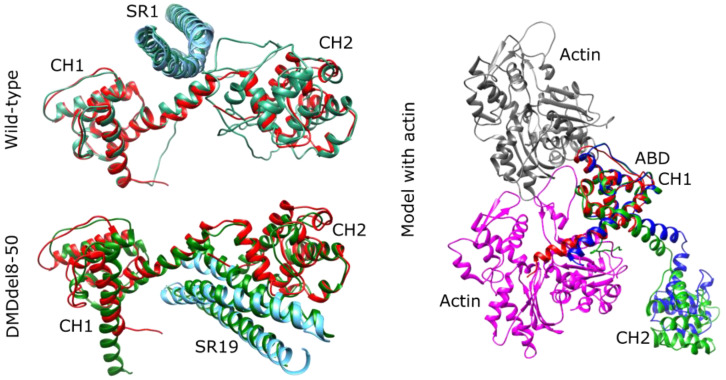
Dystrophins from DMDdel8-50 mice and wild-type mice have identical 3D structures for their actin-binding domains. (**Left**) 3D alignment of AlphaFold-predicted structure of the dystrophin Δ8–50 fragment with human wild-type dystrophin fragments taken from PDB 1DXX and 3UUN. The Δ8–50 fragment includes the actin-binding domain, a fragment of hinge 3, and full spectrin-like repeat 19 (first 357 amino acids are shown); the wild-type dystrophin fragment includes the actin-binding domain, hinge 1, and full spectrin-like repeat 1 (first 449 amino acids are shown). Red is for chains A of dystrophin ABD from 1DXX; green is for AlphaFold simulation; cyan is for spectrin repeat 1, chain A from 3UUN. (**Right**) 3D alignment of predicted AlphaFold models for two CH domains in open conformation for wild-type (green) and DMDdel8-50 (blue) variants with ABD domain (red, PBD: 6ANU, chain a). Two actin domains (PDB: 6ANU, chain A and D) are provided in pink and grey, respectively. See also Supplementary Videos S1–S3.

The structures of the CH domains and spectrin domain in both the wild-type and BMDdel8-50 variants closely matched those available in the PDB (Figure 6), with RMSD values of less than 1.75A. We then assessed whether the exon skipping affected the actin-binding properties of the CH1 domain. By superimposing the CH1-CH2 structures onto the ABD from the cryoEM actin-ABD structure complex (PDB: 6ANU), we found that both the wild-type and BMDdel8-50 CH1 domains aligned perfectly with the ABD structure from the 6ANU file, indicating that the BMDdel8-50 variant can still bind to actin through the CH1 domain (Figure 6). In the open conformation, the CH2 domain did not produce any sterical conflict with actin.

The orientation of the two CH domains and the following spectrin domain varied among the five models generated by AlphaFold for each prediction. This is due to the role of the hinge domain, which provides flexibility to the protein chain and allows for different orientations based on the protein’s binding partners in the cell, rather than being a consequence of the protein structure. According to the findings, truncated dystrophin binding to actin should be unaffected by the absence of a portion of CH2.

## 3. Discussion

In this study, we created a new mouse model with an in-frame deletion of exons 8–50 in the *DMD* gene. Despite shortened dystrophin’s correct localization on the sarcolemma and a significant level of expression in skeletal muscles, the heart, and the diaphragm, mice exhibited the muscular failure characteristics of DMD. Additionally, we observed an increase in the quantity of small and central nuclear myofibrils, which often accompany progressive muscular dystrophy, as well as a decrease in the presentation of DAGC proteins. Pathogenic in-frame deletion of exons 8–50 has been reported in LOVD (ID #00286638). The patient, who is now 14 years old and nonambulatory, was born in a nonconsanguineous family after a normal pregnancy and labor. The motor delay was characterized by sitting with support at 1.5 years old, standing with support at 4 years old, walking with frequent falls at 4.5 years old, and drawing at 6 years old. He was never able to run or write. The patient had ankle contractures and mild calf muscle hypertrophy. Additional tests revealed CK levels of 5786 U/L and a myopathic pattern on EMG. There were no other issues with feeding, vision, hearing, speaking, or social activity. Dystrophin levels and patterns of expression were not investigated (Dr. Shubha Phadke, personal communication). In accordance with patient data, the mouse model represents DMD symptoms on the background of in-frame deletion of exons 8–50, constituting an exception to the “reading-frame rule”.

The first possible explanation for this exception is a low amount of truncated protein expression. In the created mouse model, shortened dystrophin expression varied between 6.8 and 21% in different muscle tissues. Some reports demonstrate that 4% dystrophin is sufficient for improved muscle function and survival [33,34,35]. Other reports show that 20% dystrophin improves symptoms [36], and 15% could be sufficient to protect against contraction-induced injury in DMD model mice, but more than 40% dystrophin is needed to also improve muscle force [37]. Another paper describes BMD model mice expressing 30–50% and exhibiting susceptibility to contraction-induced injury and harboring motor function deficits [38]. According to a recent review, 20% equally distributed dystrophin is sufficient for protection from disease progression [39]. At the same time, a correlation analysis by Van den Bergen and colleagues revealed no link between dystrophin levels and disease progression. The authors claim that the course of the disease is determined by the breakpoint of the deletion rather than the level of truncated dystrophin expression, which exceeds 10% [6]. Nicolas and colleagues have also provided evidence that the development of the disease in BMD patients appears to be influenced by the particular structure of dystrophin rather than the quantity of protein [24]. We came to the conclusion that the lowered dystrophin levels in the muscles of DMDdel8-50 mice are a contributing factor to the severity of the disease. It does not appear to be the only cause of the DMD phenotype, however.

Another explanation for exceptions to the “reading-frame rule” is the insufficient length of truncated dystrophin. Fanin and colleagues calculated the deletion size threshold effect. They claimed that dystrophins with fewer than 43 exons and approximately 200 kDa produce DMD rather than BMD [7]. Several investigations have revealed that large *DMD* deletions (>36 exons) result in a severe phenotype [40,41]. In addition, Nevo and colleagues described three examples of large in-frame deletions leading to DMD with a normal level of shortened proteins [42]. Similarly, Winnard and colleagues described a patient with an in-frame transcript lacking exons 3–41. Despite the expression of 200 kDa protein at a level of 84% of normal by WB and normal dystrophin localisation by IHC, this patient was diagnosed with DMD [40]. The overall length of the shortened dystrophin of BMDdel8-50 mice was 168.5 kDa, as opposed to 427 kDa for normal dystrophin. The introduced deletion removed 42 exons, resulting in a transcript with 37 exons out of 79. The observed DMDdel8-50 mice phenotype may be explained by the fact that the produced dystrophin does not achieve the minimum required size. Nonetheless, there are examples of relatively short microdystrophins that, when delivered by viral vectors, can prevent disease progression. For example, the weight of the R4–R23/71–78 microdystrophin employed in preclinical and clinical trials by Sarepta Therapeutics was 138 kDa (Figure 1B) [43]. Its administration improved the histologic and strength properties of *mdx* mice and DMD patients’ muscles [43,44]. In comparison to the R4–R23/71–78 microdystrophin, which has a length of roughly 1200 aa, the 8–50 dystrophin has a length of 1465 aa. The CT domain, which is missing in this microdystrophin, is made up of more than 300 amino acids from the 8–50 dystrophin. Given that the CT domain is nonobligate, the “effective” length of tDp427m dystrophin in the absence of exons 8–50 is quite comparable to R4–R23/71–78 (Figure 1B), and, therefore, it is unlikely that protein length is the only factor causing the truncated dystrophin to operate improperly.

The functional properties of the resulting dystrophin may be affected by the domain composition and interactions. The connection of CH2 to the rod via the hinge corresponds to natural protein structure (Figure 1B). Nevertheless, shortened dystrophin was nonfunctional when exons 8–50 were deleted. This may highlight the significance of missing residues in the interdomain interaction. The obtained version lacks the 22 AA residues from CH2 encoded by exon 8 and the 10 AA residues from H3 encoded by exon 50. The three-dimensional structure and formation of binding sites in N-ABD can be affected by the deletion of terminal 22AA in CH2. This is indirectly corroborated by a meta-analysis of patient data revealing that exon 8 absence at the start or end of an in-frame deletion (exon skip-equivalent mutations) was not linked with a lower incidence of DMD compared to corresponding exon skip-amenable mutations [5]. However, based on the results of in silico prediction, we did not uncover any essential spatial changes that could disrupt dystrophin–actin binding.

The significance of hinges in dystrophin functionality is increasingly garnering research interest. Since hinges do not create a well-structured spatial domain, it appears that this topic was understudied and underestimated. According to a report published in 2010, not all of dystrophin’s hinges contribute equally. Banks and colleagues found that replacing hinge II with hinge III enhanced microdystrophin’s functional capacity [45]. On the other hand, analysis of patient data showed that the presence of hinge III in truncated dystrophin was associated with a more severe form of the disease [46]. A recent study discovered that crucial dystrophin structures required for protein function contain hinge IV [47]. Consequently, shortened proteins may malfunction due to the simultaneous loss of hinges I and II or a lack of residues inside hinge III. Another component found in all microdystrophins but missing in Δ8–50 dystrophin is the first spectrin-like repeat [13]. Harper and colleagues demonstrated in 2002 [48] that spectrin-repeats from dystrophin and α-actinin are not similar. A form of microdystrophin, which contained actin-derived SR1-3, restored DAGC components on the sarcolemma and provided some protection from contraction-induced damage, but was unable to repair the *mdx* mouse’s morphological abnormalities. This finding supports the idea that SR1 is required for dystrophin to function properly.

To summarize, we suggest that the combination of a large deletion, a disrupted interdomain sequence, and the absence of critical domains is causing truncated protein dysfunction in DMDdel8-50 animals. Apart from the quality of the dystrophin, the extent of dystrophin cannot be ruled out as a contributing factor to disease severity.

In contrast to the numerous DMD animal models, there are comparatively few BMD models. So far, one rat and one mouse model have been reported with an in-frame deletion in the *DMD* gene [38,49]. When compared to *mdx52* knockout mice, a mouse model with exons 45–47 deleted (bmx) revealed much reduced skeletal muscle damage [38]. In contrast, the IF rat model with exon 3–16 deletion displayed muscle degeneration consistent with DMD [49]. Simultaneously, rats did not differ substantially from control mice of the same age in terms of muscle-strength parameters. The bmx mice outperformed their KO counterparts (*mdx52*) but fell short of wild-type animals on a variety of strength and cardiac function tests and had higher CK levels.

Along with the acquired DMDdel8-50 mice, previously established animal models with in-frame DMD mutations mirror the variability seen in BMD patients. Their comprehensive examination could aid in understanding the diversity of phenotypes. BMDdel8-50 mice and IF could serve as an excellent model for preclinical testing of gene replacement and other therapies intended for patients with severe disease progression in the presence of dystrophin expression.

The Dp71 protein was found in the skeletal muscles of DMDdel8-50 mice, but not DMDdel8-34 mice, similar to previously obtained in-frame models [38,49]. Dp71 upregulation was also found in DMD patients’ muscle samples [50]. Dp71 is known to be widespread, with a high prevalence in nervous tissue. It has been shown that expression in mature skeletal muscles causes inflammation and DMD-like pathology [51]. The role of the Dp71 dystrophin isoform in muscular dystrophy progression and the mechanisms underlying its upregulation in the presence of truncated dystrophin remain unclear. In almost all tests, DMDdel8-50 mice did not differ from DMDdel8-34 KO animals. Only during eccentric contraction did we find a higher force generated by isolated TA muscles (Figure S9) and a higher incidence of small myofibers (Figure 4B). Grip test results in adult mice deteriorated when compared to juvenile mice, which also distinguishes mice with in-frame and out-of-frame mutations (Figure 4C). Possibly, Dp71 overexpression can be responsible for these differences.

In conclusion, the phenotype of DMDdel8-50 mice shows that deletion of exons 8–50 in the DMD gene does not halt or prevent disease progression and cannot be considered as a potential therapy.

## 4. Materials and Methods

### 4.1. Guide Design and Synthesis

Staphylococcus pyogenes Cas9 guides were designed for *DMD* (GenBank ID: NC_000086.7) intron 50 in the CHOPCHOP online tool [29] and selected based on minimal predicted off-target activity. Synthetic oligos were obtained from Evrogen (Moscow, Russia). For sgRNA synthesis, two partially complementary oligonucleotides were used: sgR, encoding the T7 promoter, and sgF, encoding the guide RNA sequence. For template amplification, oligos were combined in an equimolar ratio and polymerized with Pfu-polymerase (Promega, Madison, WI, USA) in a T100 thermocycler (Bio-Rad, Hercules, CA, USA) using the following program: 95 °C for 1 min; 30 cycles of 95 °C for 30 s, 65 °C for 30 s, and 72 °C for 30 s; and 72 °C for 5 min. To purify the template from the reaction mix, the Clean Up kit (Evrogen) was used, and the sgRNA was then transcribed from the T7 promoter in vitro using the RiboMAX Express kit (Promega). Further purification was performed by phenol–chloroform extraction followed by isopropanol precipitation. Pure sgRNAs were dissolved in nuclease-free water. We determined the concentration using the Qubit RNA HS kit (Thermo Fisher Scientific, Waltham, MA, USA) and the sgRNAs were stored at −70 °C before use. The Cas9 expression construct pET28a/Cas9-Cys, a gift from Hyongbum Kim (Addgene plasmid #53261), was linearized and transcribed in vitro using the mMessage mMachine T7 kit (Ambion, Austin, TX, USA). Post-synthesis steps were identical to sgRNAs. For microinjection, guide RNA (20 ng/µL) was mixed with Cas9 mRNA (50 ng/µL) in TE buffer (10 mM Tris-HCl (pH 7.4); 0.1 mM EDTA). The components were mixed immediately before microinjection, heated up to 65 °C for 5 min, and chilled on ice. We also used Cas9 Nuclease (NEB) to test the freshly synthesized sgRNA activity in vitro. As a template for the new guides, target regions amplified from murine genomic DNA were used. Analysis was performed according to manufacturer recommendations.

### 4.2. Microinjections into the Pronucleus of Zygotes, Embryo Cultivation, and Transfer

In 3-week-old immature female mice (C57BL/6×CBA) weighing 12–13 g, superovulation was induced by administering 5 units of pregnant mare serum gonadotropin (Folligon; Intervet International, Boxmeer, The Netherlands) and 5 units of human chorionic gonadotropin (hCG, Pregnil; N.V. Organon, Jersey City, NJ, USA) 46–48 h later. After injection, the female mouse was immediately placed in a cage with a male mouse for mating. Ovulation occurred within 11–14 h after hCG injection. Within 12–13 h after copulation, i.e., 25–27 h after hCG injection, superovulated mice were sacrificed by cervical dislocation, and oviducts were surgically removed and placed into the M2 medium. Then, oviducts were dissected and COCs (cumulus oocyte complexes) were obtained. COCs were treated by hyaluronidase (Lidase; Microgen, Moscow, Russia) to clean the cumulus cells. Hyaluronidase was added into the M2 medium to achieve the final concentration of hyaluronidase of 25 IU/mL. Then, embryos were flushed in the M2 medium three times. The two pronuclear-stage zygotes were placed in a chamber consisting of two coverslips, with one fixed above the other so that the upper and lower droplet edges of the M2 medium were flat and parallel. Pronuclei were visualized using a differential interference contrast microscope (Axiovert 200; Carl Zeiss, Oberkochen, Germany). The microinjection needles were made from G100 glass capillaries (Narishige, Tokyo, Japan) on a P97 puller (Shutter Instruments, Novato, CA, USA), and holder capillaries were made from GD1 capillaries (Narishige) on a PC-10 puller (Narishige) and MF-900 Microforge (Narishige). The genome editing complex was microinjected into the cytoplasm of mouse zygotes. After microinjection, the zygotes were transferred into the bicarbonate-buffered medium (Drobleniye; Pan-Eco, Moscow, Russia) for 2–3 h in a CO_2_ incubator (150 IGO; Thermo Electron Corporation, Waltham, MA, USA) at 5% CO_2_/95% air and 100% humidity, and embryos were cultured in 600 μL of medium and then assessed visually; embryos without fragmentation, and of round shape, were transferred to recipient mice. To obtain recipient pseudopregnant mice, CD1 females were bred with vasectomized CD1 males overnight. Mice with an observed copulative plug in the morning were used as recipients. For embryo transfer, recipients were anesthetized by the simultaneous intraperitoneal injection of Xyla (Interchemie, Castenray, The Netherlands) at a dosage of 2 mg per kg and Zoletil (Virbac, Carros, France) at a dosage of 20 mg per kg. Then, embryos were surgically transferred into the oviducts at the rate of 10 embryos per oviduct.

### 4.3. Animal Studies

DMDdel8-50 mice, dystrophin-deficient DMDdel8-34 mice, and their C57BL6/CBA background mice were bred in a vivarium of IGB RAS. All mice were maintained under a controlled 12-h photoperiod, and water and food were provided ad libitum.

### 4.4. Creatine Kinase

Serum for analysis was derived from blood collected from the jugular veins. Blood was left for 15 min at room temperature to promote clot formation and then centrifuged at 3500× *g* for 10 min at 4 °C. Then, the serum was collected. CK activity was determined using the Creatine Kinase Activity Assay Kit (DiaVetTest, Pushchino, Russia) and a NanoPhotometer (Implen, Westlake Village, CA, USA).

### 4.5. Histopathology

The organs were weighed, fixed in 10% buffered formalin, and embedded in paraffin. Five-micron-thick transverse sections were stained with hematoxylin and eosin (H&E) according to the routine procedure described previously [52]. Images were acquired with a Nikon Eclipse Ti-E at 200× magnification.

### 4.6. Hanging Wire Test and Grip Strength Test

Functional tests were performed for each mouse according to the protocol described by Aartsma-Rus and van Putten [53]. For the hanging wire test, a 55 cm-wide, 2 mm-thick metallic wire was installed 37 cm above a layer of bedding. Each mouse was granted three trials to hang on the wire by the forelimbs with a 30 s recovery period between trials. The maximum hanging time for the three trials was recorded and used as an outcome measurement. Grip strength was quantified using the Grip Strength Test Meter for Mice and Rats (IITC Life Science Inc., Woodland Hills, CA, USA). Grip measurements were replicated 5 times each session, with intervals at a minimum of 1 min between replicates, and mean values were used for the analysis.

### 4.7. Measurement of Isometric Force and Muscle Susceptibility to Eccentric Contraction-Induced Injury

Measurements of isometric muscle force and susceptibility to eccentric contraction-induced injury were performed for each mouse at the end of the experiment according to the protocols described earlier [54]. First, the tested muscle was adjusted to an optimum length (Lo) to produce the maximum tetanic force. Then, while held at Lo, the muscle was stimulated every 2 min at increasing frequencies (10 to 250 Hz) to generate force frequency curves to obtain the maximal tetanic force (Po). Then, both Lo and muscle mass were recorded and used to normalize to the physiological cross-sectional area CSA ([Lo × density]/mass) and calculate the specific tetanic force (sPo) based on previous protocols (see SOP DMD_M.2.2.005). In the eccentric contraction protocol, the force deficit at each lengthening contraction (P1-P7, P1 = Po) was calculated as (Po-P2-7)/Po × 100%.

### 4.8. Genomic DNA Isolation, PCR, and Sequences Analysis

For mice genotyping, tail biopsies were collected at the age of 2 weeks. Crude DNA samples were prepared using brief alkaline lysis; a small tail biopsy fragment was lysed in an alkaline lysis buffer [25 mM NaOH, 0.2 mM Na2-EDTA (pH 12)] for 1 h at 95 °C and neutralized with 40 mM Tris-HCl (pH 5), and 2 μL of the resulting solution was used as a template for PCR. Primers dmd_sg31_434F and SpSg50in1-626R flanking deletion were used to identify BMDdel8-50 mice. For other PCR protocols, genomic DNA was purified from frozen tissue samples using the DNeasy Blood & Tissue Kit (Qiagen, Hilden, Germany) according to the manufacturer’s protocol. The concentration was estimated using a NanoPhotometer (Implen). Guide recognition sites in introns 7 and 50 of the DMD gene, the deletion breakpoint, and the top 3 off-target sites (Table 3) predicted in the Benchling Biology Software (2023) [55] were amplified with the primers listed in Table S1. We used a Gene Pack PCR-core kit (Isogene, Russia) or Phire Tissue Direct PCR Master Mix (Thermo Scientific) for amplification. After Sanger sequencing, we analyzed the raw data in the Benchling Biology Software (2023) [55]. For the quantitative analysis of indel presence in OT sites, we used the Tracking of Indels by DEcomposition (TIDE) analysis [56]. For TIDE analysis of OT sites, guide RNA was entered with corresponding replacements (Table 3). The indel size ranges were set to 10 nucleotides upstream and downstream of the predicted cut site. A *p*-value less than 0.01 was accepted as significant.

### 4.9. RT–qPCR

The total RNA was purified from cells or frozen tissue samples using TRI Reagent (MRC) following the manufacturer’s recommendation. RNA was additionally treated with DNase I (NEB), and cDNA synthesis was performed using a Magnus reverse transcriptase (Evrogen) and a mix of random and oligo(dT) primers 1:1. PCR for detecting native and shortened cDNA fragments was performed using Mm_dmd_142F for exon 3 and 4 junctions and DMD51ex-502Rev for exon 51 (Table S1). PCR with Phire Tissue Direct PCR Master Mix (Thermo Scientific) was performed using following amplification program: 98 °C for 5 min; 35 cycles of 98 °C for 15 s, 64 °C for 15 s, and 72 °C for 90 s; 72 °C for 5 min. Dp427 transcripts were amplified by primers Mm_dmd-187F and Dmd_mus_ex5-81R; primers were designed to amplify the 5′ region preceding mutation and genome editing sites. The following qPCR conditions were used: 94 °C for 3 min, followed by 40 cycles of 94 °C for 15 s, 66 °C for 15 s, and 72 °C for 15 s (+fluorescence measurement). Primers and probes specific to the reference genes *Rpl13a*, *Ap3d1*, and *Csnk2a2* (Ta = 55 °C) were used to normalize the gene expression in mice [57].

### 4.10. Western Blotting

Frozen muscle samples were homogenized in RIPA buffer (50 mM Tris pH 8.0, 150 mM NaCl, 1% Triton X-100, 0.5% sodium deoxycholate, 0.1% SDS, 0.01% protease inhibitor cocktail) in a Tissuelyser LT (Qiagen). The total protein concentration was measured by a BCA Protein Assay Kit (MilliporeSigma, Burlington, MA, USA) on Clariostar Plus (BMG Labtech, Ortenberg, Germany). Equal amounts of lysates (up to 20 mkg of total protein) were mixed with 4x Laemmli sample buffer, incubated at 95 °C for 5 min to denature proteins, and resolved in 4–15% Mini-PROTEAN^®^ TGX™ Precast Protein Gels (Bio-Rad). Proteins were transferred to a PVDF membrane (Bio-Rad) using a standard wet transfer protocol in a Mini Trans-Blot Cell (Bio-Rad). Nonspecific binding was blocked by incubating the membrane in 5% dry milk in TBST for 1 h. For dystrophin detection, we used rabbit polyclonal antibodies (ab15277, 1:400; Abcam, Cambridge, UK). Rabbit monoclonal antibodies to α-sarcoglycan (ab189254, 1:2000; Abcam) and mouse monoclonal antibodies to β-dystroglycan (NCL-b-DG, 1:200; Leica Biosystems, Wetzlar, Germany) were used for DAGC components identification. GAPDH was used as a loading control (ABS16, 1:3000, MilliporeSigma). Goat anti-rabbit IgG (H + L) cross-adsorbed secondary antibody HRP (G-21234, 1:10,000; Invitrogen, Waltham, MA, USA) and goat anti-mouse IgG (H + L)-HRP (1706516, 1:1500; Bio-Rad) were used as secondary antibodies. Incubation was carried out overnight at 4 °C with primary antibodies and for 1 h at room temperature with secondary antibodies. Signal detection was performed using the Clarity Western ECL Substrate (Bio-Rad). Detection and quantification were performed using the iBright FL1500 Scanner (Thermo Fisher) and iBright analysis software (4.0.1).

### 4.11. Immunofluorescence

Tissues for staining were embedded in Tissue-Tek OCT Compound mounting medium (Sakura Finetek, Tokyo, Japan) and frozen in isopentane (PanReac, Barcelona, Spain) precooled with liquid nitrogen. Ten-micron-thick transverse sections were obtained on a CM 1510-1 cryostat (Leica). Muscle sections were fixed in 4% paraformaldehyde (PFA; AppliChem, Darmstadt, Germany) and 2% D(+)-Sucrose (AppliChem) in PBS for 30 min at room temperature and permeabilized in PBST (0.1% Triton X-100 solution in PBS). After permeabilization, nonspecific antibody binding was blocked with 3% bovine serum albumin (BSA; PanEco, Russia) solution in PBST for 1 h at room temperature. Sections were incubated with rabbit primary antibodies that recognize the dystrophin C-terminus (ab15277, 1:300; Abcam) or anti-α-Sarcoglycan rabbit monoclonal antibodies (ab189254, 1:2000; Abcam), and co-stained with rat monoclonal antibodies raised against laminin 2α [4H8-2] (ab11576, 1:250; Abcam) at 4 °C overnight. Antibodies were diluted in a 3% BSA blocking buffer. The following day, the slides were washed and then incubated with Alexa Fluor 488-labeled goat anti-rabbit (ab150077, 1:1000; Abcam) or Alexa Fluor 633-labeled goat anti-rat secondary antibodies (A21094, 1:500; Invitrogen) for 1 h at room temperature. The cryosections were washed with PBST between each stage. Nuclei were counterstained with Hoechst 33342 (1:1000; Thermo Fisher). Stained sections were mounted in ProLong Gold antifade mounting medium (P36930; Invitrogen). Appropriate negative tissue and isotype controls were implemented in the experiments. Fluorescence images were captured on Zeiss LSM 880 and Leica Stellaris 5 confocal microscopes and analyzed in Fiji [58]. Quantitative analysis of dystrophin and α-sarcoglycan staining intensity was performed using the CellProfiler 4.2.1 software [59]. Separate steps of intensity quantification are demonstrated in Figure S5. The percentage of myofibers with centrally located nuclei and the Feret diameter were determined from transverse sections of laminin- and DAPI-stained muscles on five random fields of view per section per mouse.

### 4.12. Dystrophin Protein Modeling

To verify the absence of the wild-type structure of mouse dystrophin in the AlphaFold database [60], we used the Uniprot code P11531. Using the standalone version of AlphaFold [60] with default parameters, we modeled the N-terminal fragments of both the wild-type and Δ8–50 mutant variants. In the monomer mode, we modeled the wild-type structure from amino acid residues 1–449, while for the Δ8–50 variant, we modeled a sequence covering residues 1–357, which consisted of residues 1–216 and 2431–2570 of the wild-type sequence, with alanine in between. In the multimer mode, we added a second chain covering amino acid residues 1–246 for the wild-type and amino acid residues 1–216 for the Δ8–50 variant. In all cases, out of five predicted structure models, we used the relaxed model with the highest confidence score. We drew pictures with Chimera [61].

### 4.13. Statistical Analysis

Statistical analysis of the results was performed using the GraphPad Prism 9 software. Differences identified between groups were determined using an ordinary one-way ANOVA with Dunnett’s test or the Kruskal–Wallis test with Dunn’s test for multiple comparisons. Data in the text, tables, and graphs are presented as mean ± standard deviation (SD) or standard error of mean (SEM). Differences between the control DMDdel8-34 group and those with *p* < 0.05 are considered significantly different.

## Figures and Tables

**Table 1:** Guide RNA characteristics.

ID	*DMD* Intron	Sequence, 5′-3′ (PAM)	Coordinates
sg31 ^1^	7	GTGGCAGACTAGTAGTTTG(AGG)	X:82534746-82534764
sg50in1	50	GGTTAGTAGCTCTCCATCA(GGG)	X:83480495-83480513
sg50in2	50	GACATCGGCACAACAATCA(AGG)	X:83504960-83504977
sg50in3	50	GGTCCAAACCTATCTGTGA(GGG)	X:83488341-83488359

Note: ^1^ sg31 guide design was previously reported in [26].

**Table 2:** Embryo microinjection and transfer statistics.

Deletion	Guide Combination	Embryos	Transplanted	Recipients	Newborn Pups	Founders
8–50	sg31 + sg50.3	106	28	2	7	0
8–50	sg31 + sg50.1	330	67	6	10	2 + 2 ^1^

Note: ^1^—died immediately after birth.

**Table 3:** Potential off-target site sequences.

Site	Sequence, 5′-3′ (PAM)	Chr
Sg50.1	GTTAGTAGCTCTCCATCA (GGG)	X
Sg50.1 OT1 (Asb18)	GTTtaTAGCTCTCCATCA(TGG)	1
Sg50.1 OT2 (Malrd1)	GTaAGTAGCTCTtCATCA(AGG)	2
Sg50.1 OT3 (Ift88)	aTTAGTAGCTCTCCAcCA(AGG)	14
Sg31 target	GTGGCAGACTAGTAGTTTG(AGG)	X
Sg31 OT1 (*Tff3*)	cTGcCAGACTAcTAGTTTG(GGG)	17
Sg31 OT2 (*Unc5c*)	cTGGCAGACTAaTAGcTTG(AGG)	3
Sg31 OT3 (*Fmn2*)	tTGGaAtACTAGTAGTTTG(TGG)	1

## Data Availability

All other data are included in the article or Supplementary Information and available from the authors on request. Newly created materials are available from the authors on request.

## Supplementary Materials

The supporting information can be downloaded at: https://www.mdpi.com/article/10.3390/ijms24119117/s1.
