# Supplementary material for: In-Frame Deletion of Dystrophin Exons 8–50 Results in DMD Phenotype
Source: Int J Mol Sci. 2023 May 23;24(11):9117. doi: 10.3390/ijms24119117 (PMC10252864; doi:10.3390/ijms24119117)
Supplement: Supplementary file 1 [file ijms-24-09117-s001.zip › Supplementary information.pdf]

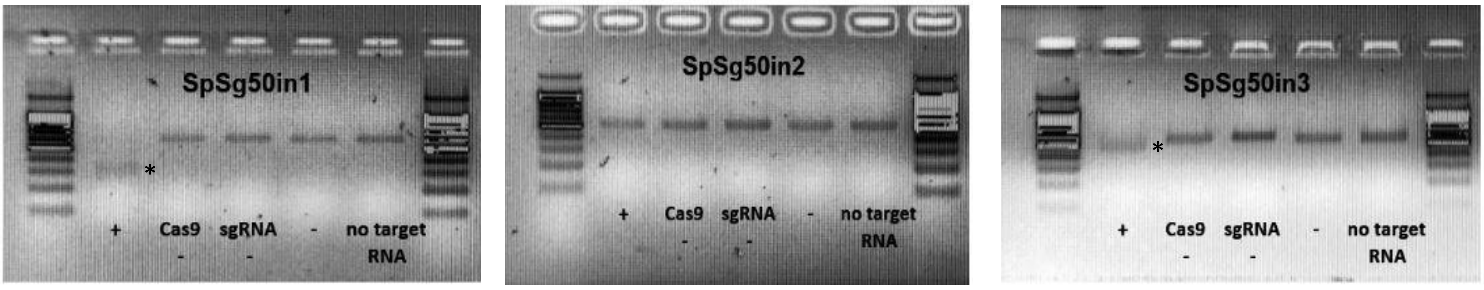

Figure S1. In vitro evaluation of RNA-guides for double-strand break introduction into intron 50. The genomic DNA surrounding the cut site was amplified and used as a template for the cleavage. The test well is marked with a "+." Negative controls without Cas9 protein and guide RNA, as well as non-targeting RNA-guide, are shown in the following wells. Cleavage products from the DNA ladder NL002 (Evrogen) were marked with an asterisk.

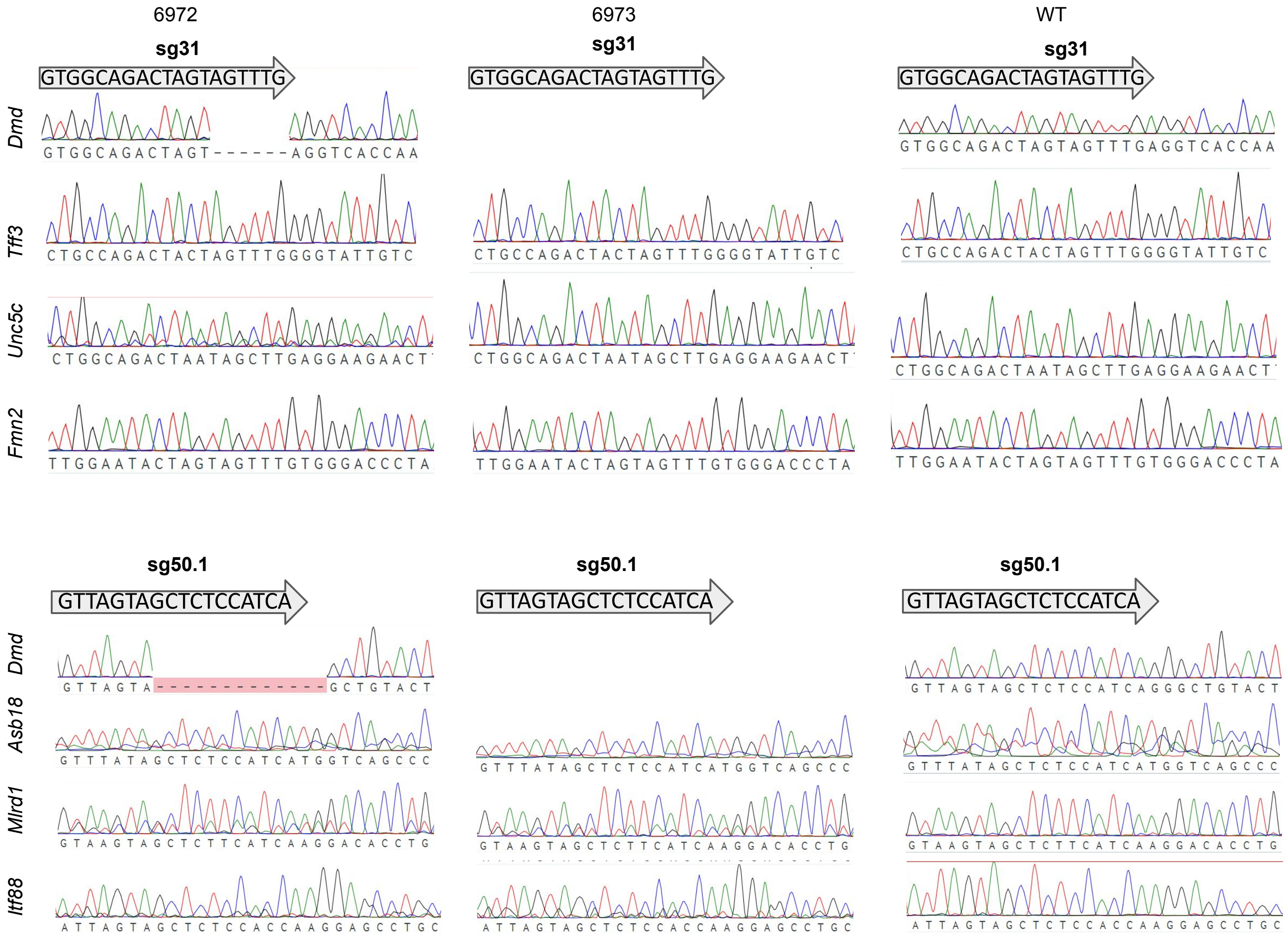

Figure S2. Sanger sequencing results for 6972 and 6973 founder mice's target (second X-chromosome in addition to desired deletion) and off-target sites.

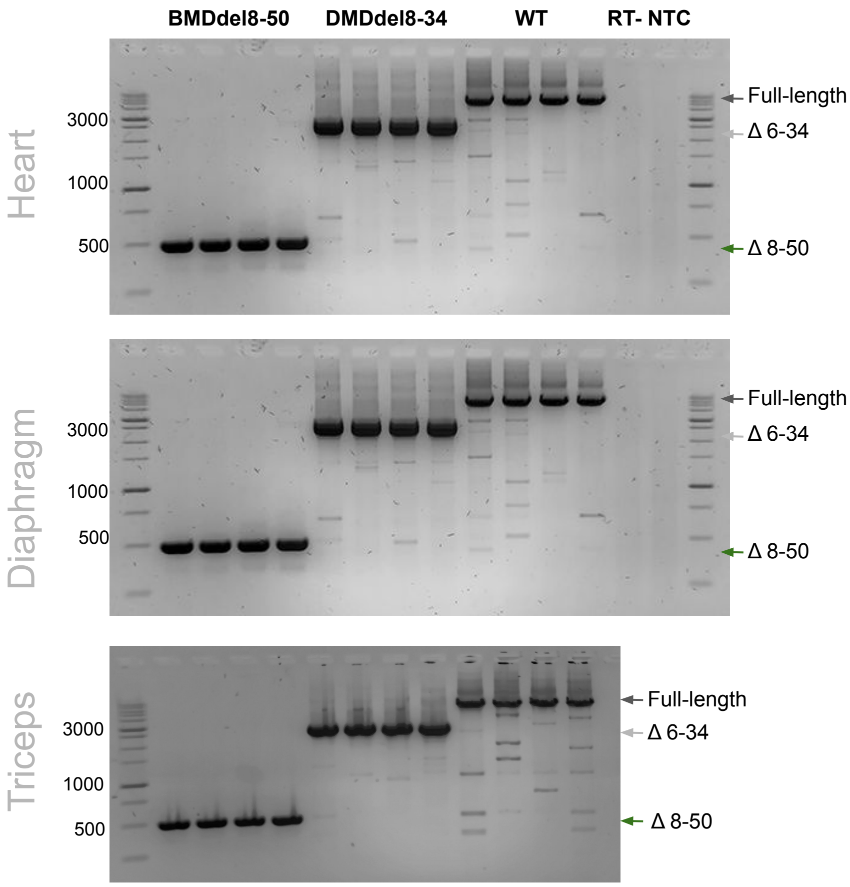

Figure S3. Truncated Dp427 transcript expression in the heart, diaphragm, and triceps from the *Dmd* gene with in-frame deletion of exons 8-50. RT-PCR analysis was carried out with primers from exons 4 and 51.

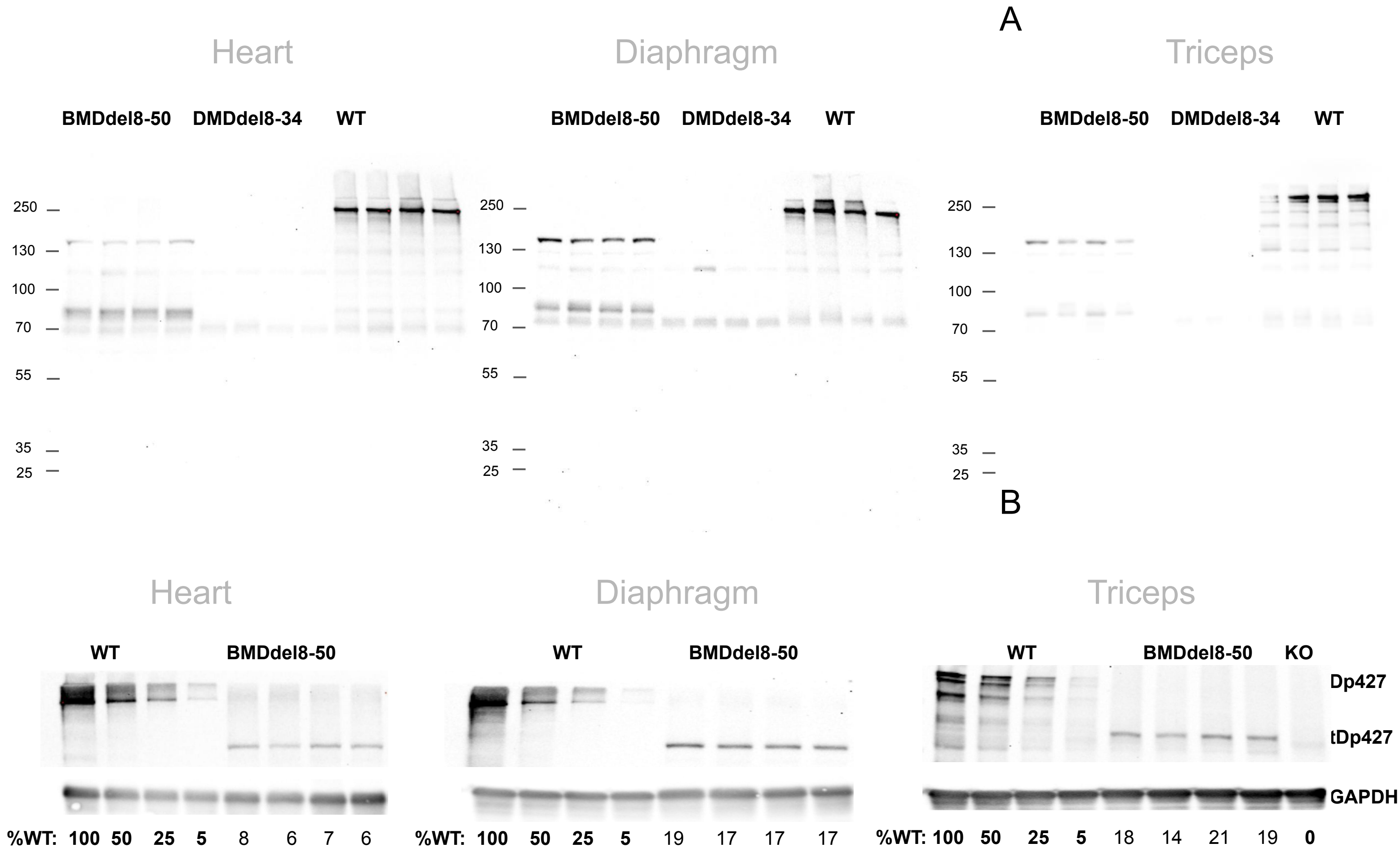

Figure S4. Western blot analysis of dystrophin expression in the heart, diaphragm, and triceps of BMDdel8-50 mice versus the DMDdel8-50 model and wild-type animals. A. Full membranes are depicted. B. Quantitative analysis of tDp427m dystrophin expression in the muscles of BMDdel8-50 mice.

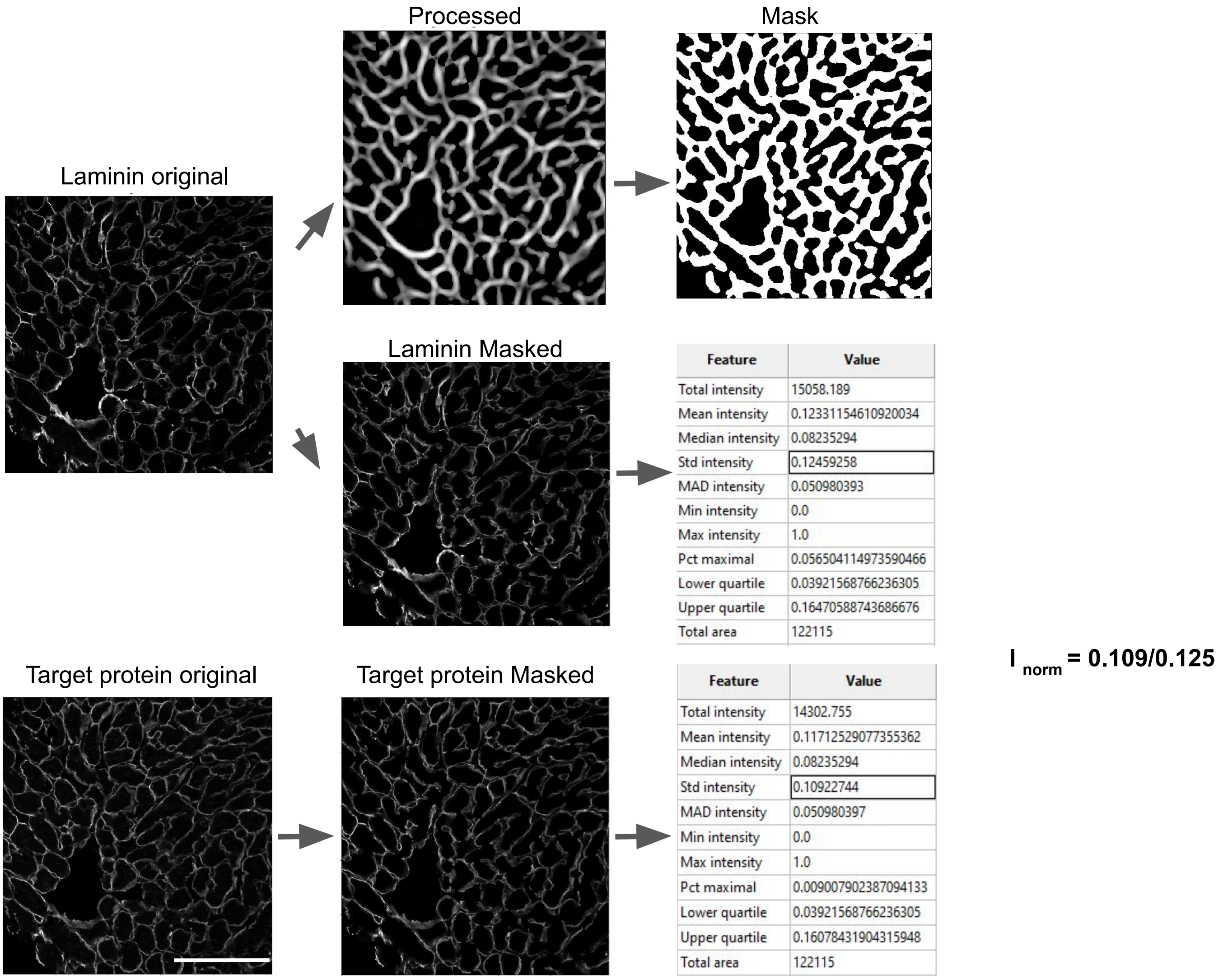

Figure S5. Separate steps from the image processing pipeline in the cell profiler software. Normalized fluorescence intensity of membrane staining with specific antibodies was calculated. Scale bar = 100 mkm.

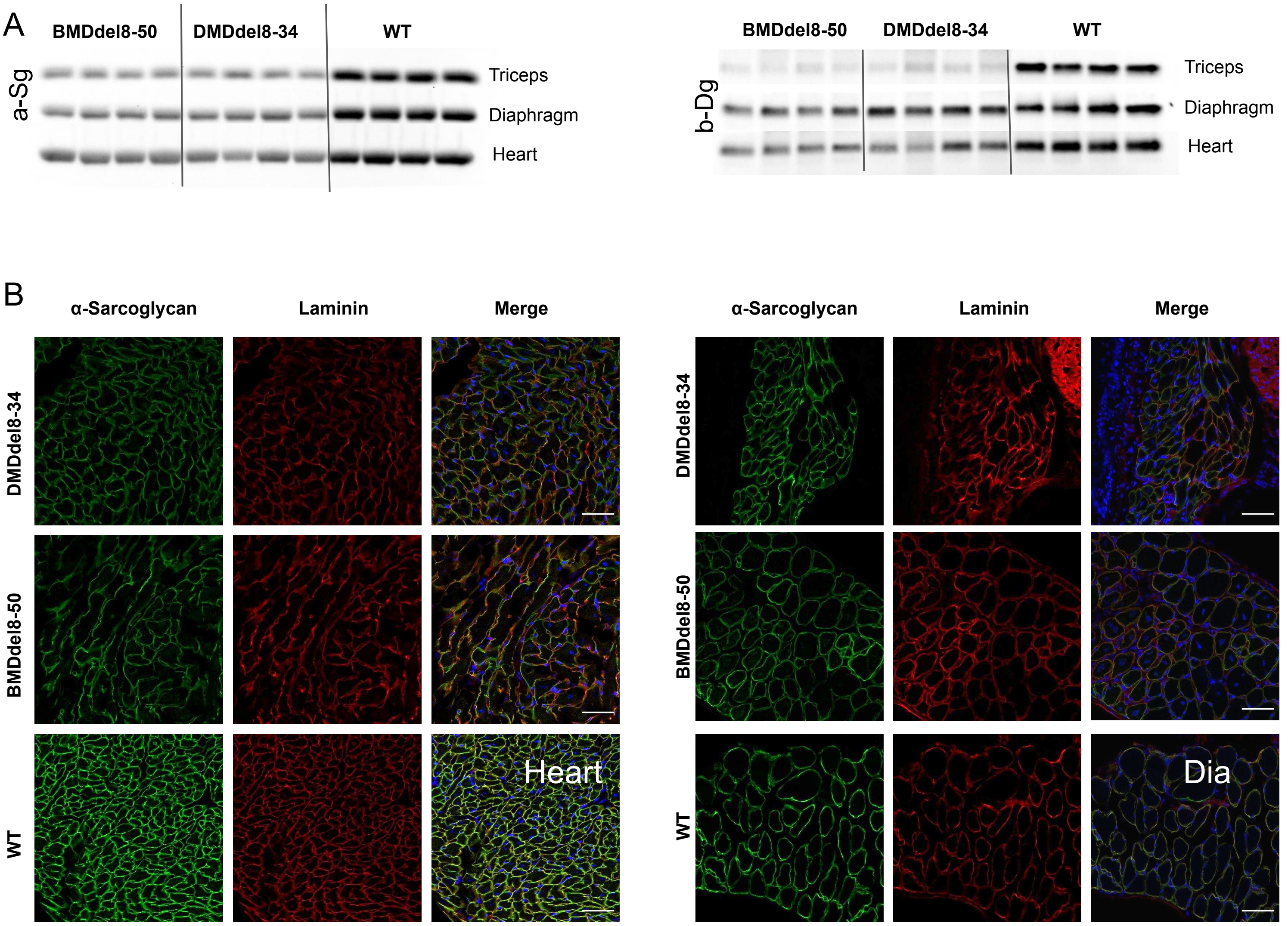

Figure S6. Analysis of DAGC components α-sarcoglycan and β-dystroglycan expression. A. Western blotting analysis of α-sarcoglycan and β-dystroglycan expression in the triceps, diaphragm, and heart. B. Immunofluorescence staining of the heart and diaphragm sections with antibodies to a-sarcoglycan. Scale bar = 50 mkm. Nuclei are contrasted with DAPI, blue.

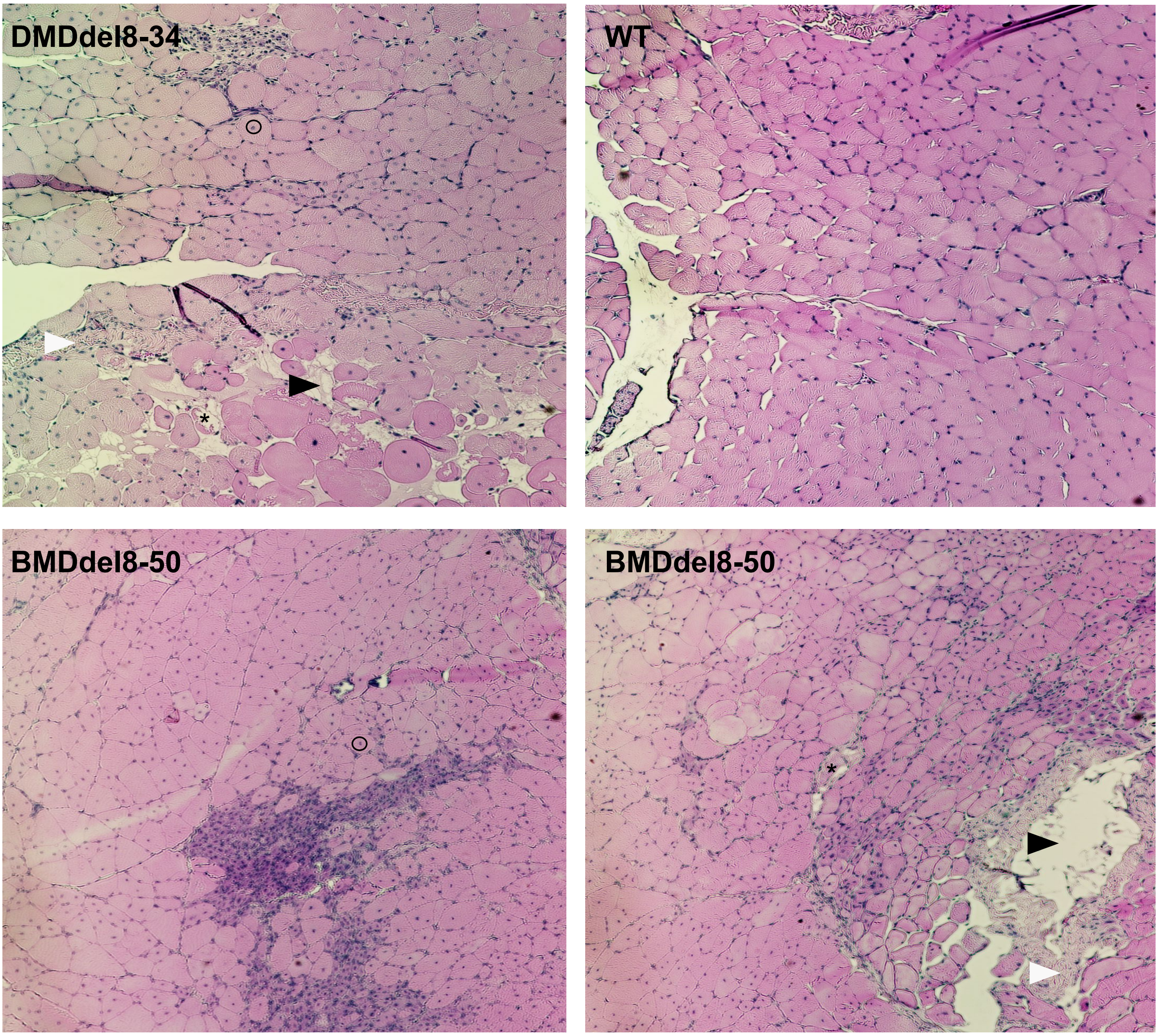

Figure S7. Gastrocnemius muscle cross-sections stained with hematoxylin and eosin. The muscle fibers of the control wild-type mice had the same diameter; nuclei are located on the periphery of muscle fibers. Both DMDdel8-34 and BMDdel8-50 mice showed disease-specific signs of inflammation, necrosis, and regeneration in skeletal muscle and the diaphragm. Central nuclei within myofiber (black circle), necrotizing myofiber (asterisk), fibrotic area (white arrowhead), and adipose tissue (black arrowhead) are demonstrated. Magnification x200.

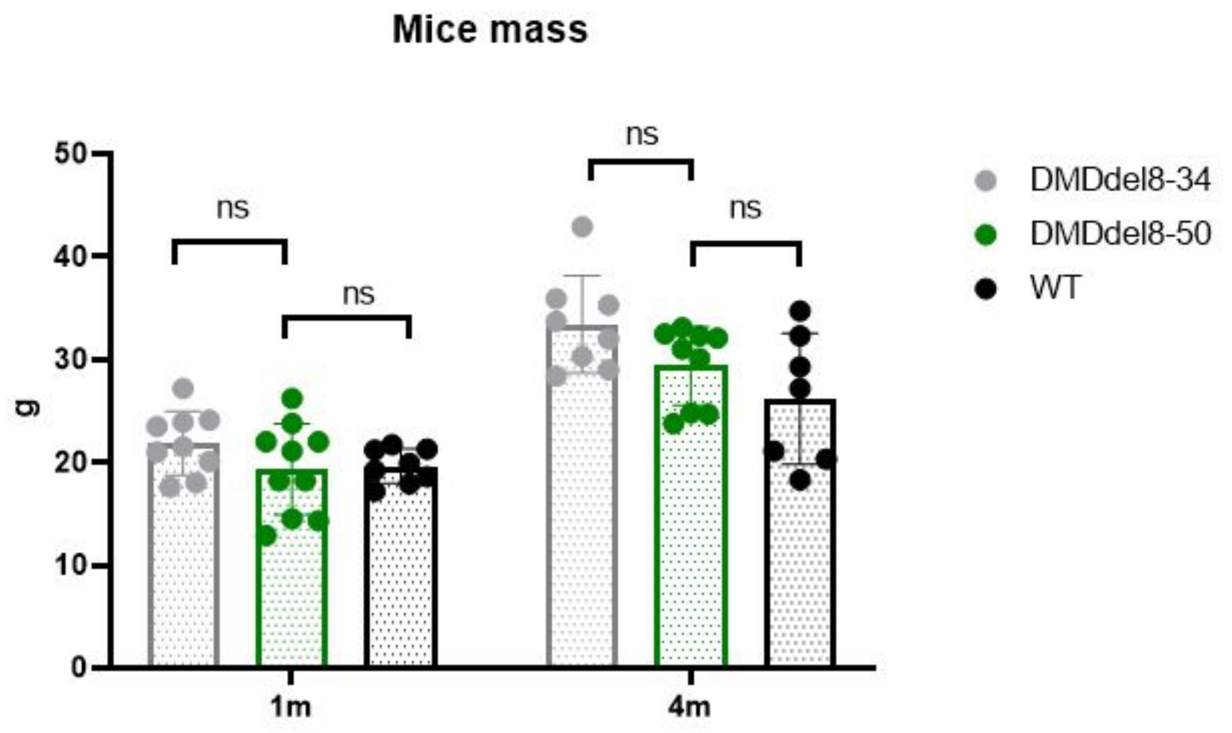

Figure S8. Mice body weight.

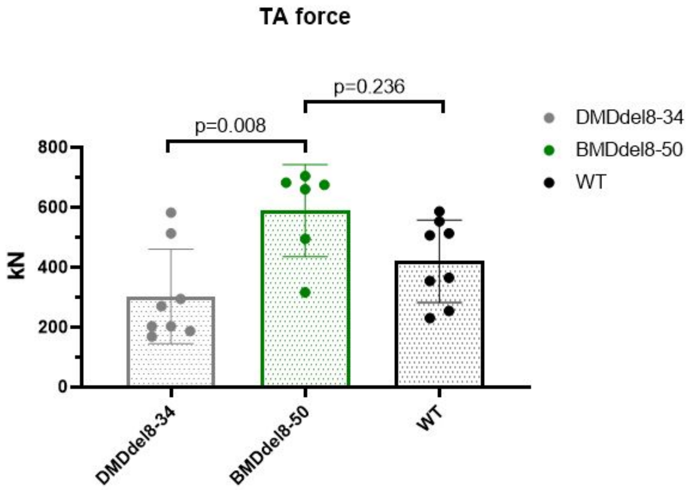

Figure S9. Force generated by isolated tibialis anterior muscle during eccentric contraction.

Table S1. Oligos and primers described in this paper.

Label	Sequence, 5'-3'
SgR	AAAAGCACCGACTCGGTGCCACTTTTTCAAGTTGATAACGGACTAGCCTTATTTTAACTTGCTATTTCTAGCTCTAAAAC
SpSg50in1	GAAATTAATACGACTCACTATAGGGGTTAGTAGCTCTCCATCAGTTTTAGAGCTAGAAATAGC
SpSg50in2	GAAATTAATACGACTCACTATAGGGACATCGGCACAACAATCAGTTTTAGAGCTAGAAATAGC
SpSg50in3	GAAATTAATACGACTCACTATAGGGGTCCAAACCTATCTGTGAGTTTTAGAGCTAGAAATAGC
sg31	GAAATTAATACGACTCACTATAGGGTGGCAGACTAGTAGTTTGGTTTTAGAGCTAGAAATAGC
RPL13A_F	CAACGGACTCCTGGTGTGAA
RPL13A_R	GTGCGCTGTCAGCTCTCTAA
RPL13A-FAM	FAM - AAAGACTGTTTGCCTCATGCCTGC - BHQ1
Ap3d1_F	GCCCAGCGTGTGGACATTAT
Ap3d1_R	GCCAGGGGTTTATCCAGGTC
Ap3a1-ROX	ROX-CACTGAGGAGATGCCAGAGAATGCTT-BHQ2
Csnk2a2_F	GGAGCTTGGGCTGCATGTTA
Csnk2a2_R	CCCAGAACCTTGGCAATTCG
Csnk2a2-VIC	VIC-TTCCACGGGCAGGACAACTATGAC-BHQ1
Mm_dmd-187F	AGGGCAAAAACTGCCAAAAGA
Dmd_mus_ex5-81R	TCCATCCACTATGTCAGTGCT
Mm_dmd-142F	AATGTCAACAAGGCACTGCG
DMD51ex-502Rev	AGATGGCATTTCTAGTTTGGAGA
dmd_sg31_434F	TCAAACAAAAGGCAGAAGAGTAAG
SpSg50in1-626R	CTCTACCACCCAGCTCCTCCC
dmd_sg31_434R	GGTCCAAAGTAGGCCTCGTA
Tff3-406F	TGCAGAGGTTTGAAGCACCA
Tff3-406R	CCTGATGGCCAAGGGATGTT
Unc5c-386F	GGGACTGGGTGTTTTTGCCT
Unc5c-386R	TCTCTGCCTCACTGTCACCT
Fmn2-265F	GGGCTCGTAGGGGTTCTTTAG
Fmn2-265R	GGCTAATGGGTACATGGTCTC
SpSg50in1-626F	AAGTGACCATGGCTTCCTGGG
SpSg50in1-626R	CTCTACCACCCAGCTCCTCCC
Sg50.1 OT1 (Asb18) - 541F	GAACACAAAGTTCCGCCCTCCC
Sg50.1 OT1 (Asb18) - 541R	CTCGTTGCATGCTTGGGGTCTC
Sg50.1 OT2 (Mlrd1) - 774F	CCACAAAGAAATGGGAGGGGGC
Sg50.1 OT2 (Mlrd1) - 774R	AAGGTCCCATCCCATAAGCCGG
Sg50.1 OT3 (Itf88) -331F	GTTAGCGCACAGGTTCTGGGTC
Sg50.1 OT3 (Itf88) -331R	TCTCCTCACTGTGCTCCACCAC
